# Expression analysis of *LIM *gene family in poplar, toward an updated phylogenetic classification

**DOI:** 10.1186/1756-0500-5-102

**Published:** 2012-02-17

**Authors:** Dominique Arnaud, Annabelle Déjardin, Jean-Charles Leplé, Marie-Claude Lesage-Descauses, Nathalie Boizot, Marc Villar, Hélène Bénédetti, Gilles Pilate

**Affiliations:** 1 INRA, UR0588 Amélioration, Génétique et Physiologie Forestières, CS 40001 Ardon, F-45075 Orléans Cedex 2, France; 2 CNRS, UPR4301, Centre de Biophysique Moléculaire, Equipe «Signalisation cellulaire et neurofibromatose», F-45000 Orléans, France

## Abstract

**Background:**

Plant LIM domain proteins may act as transcriptional activators of lignin biosynthesis and/or as actin binding and bundling proteins. Plant *LIM *genes have evolved in phylogenetic subgroups differing in their expression profiles: in the whole plant or specifically in pollen. However, several poplar *PtLIM *genes belong to uncharacterized monophyletic subgroups and the expression patterns of the *LIM *gene family in a woody plant have not been studied.

**Findings:**

In this work, the expression pattern of the twelve duplicated poplar *PtLIM *genes has been investigated by semi quantitative RT-PCR in different vegetative and reproductive tissues. As in other plant species, poplar *PtLIM *genes were widely expressed in the tree or in particular tissues. Especially, *PtXLIM1a, PtXLIM1b *and *PtWLIM1b *genes were preferentially expressed in the secondary xylem, suggesting a specific function in wood formation. Moreover, the expression of these genes and of the *PtPLIM2a *gene was increased in tension wood. Western-blot analysis confirmed the preferential expression of PtXLIM1a protein during xylem differentiation and tension wood formation. Genes classified within the pollen specific PLIM2 and PLIM2-like subgroups were all strongly expressed in pollen but also in cottony hairs. Interestingly, pairs of duplicated *PtLIM *genes exhibited different expression patterns indicating subfunctionalisations in specific tissues.

**Conclusions:**

The strong expression of several *LIM *genes in cottony hairs and germinating pollen, as well as in xylem fibers suggests an involvement of plant LIM domain proteins in the control of cell expansion. Comparisons of expression profiles of poplar *LIM *genes with the published functions of closely related plant *LIM *genes suggest conserved functions in the areas of lignin biosynthesis, pollen tube growth and mechanical stress response. Based on these results, we propose a novel nomenclature of poplar LIM domain proteins.

## Background

Plant LIM domain proteins are related to animal Cystein Rich Proteins (CRP), and contain two LIM domains characterized by the consensus sequence [C-X_2_-C-X_17_-H-X_2_-C]-X_2_-[C-X_2_-C-X_17_-C-X_2_-H] [1,2]. In animals, the CRP proteins mostly expressed in muscle tissues are involved in muscle differentiation, transcriptional regulation and actin organization [3,4]. In plant, the tobacco NtWLIM1 protein functions in the nucleus as a transcription factor regulating the expression of genes involved in lignin biosynthesis [5], and in the cytoplasm as an actin binding and bundling protein like the CRP1 protein in animal [6,7]. More recently, it has been shown that lily LlLIM1 and all Arabidopsis LIM domain proteins also regulate the actin cytoskeleton organization and dynamics [8,9].

Since the discovery of *SF3/PLIM1*, the first gene coding for a plant LIM domain protein [10,11], an increased number of *LIM *genes has been identified in a wide range of plant species including Arabidopsis, rice and poplar [1,2]. Whereas both *Arabidopsis thaliana *and *Oryza sativa *genomes contain six gene models, the *Populus trichocarpa *genome contains at least twelve *PtLIM *gene models resulting from the duplication of six ancestral genes [1]. These duplicated genes probably appeared during the "salicoid" whole-genome duplication event [1,12]. The plant LIM domain protein family has been divided into four groups: αLIM1, βLIM1, γLIM2, δLIM2 containing monophyletic subgroups differing according to their classification in plant taxonomic class or subclass and/or in their expression specificities [1]. In sunflower, tobacco and Arabidopsis, genes belonging to the WLIM1 and WLIM2 subgroups are widely expressed in plant, whereas genes belonging to the PLIM1 and PLIM2 subgroups are preferentially expressed in pollen [2,8,13]. However, the expression pattern of genes classified into the βLIM1 group and the XLIM1 and PLIM2-like subgroups remains unknown and there is no Arabidopsis and rice orthologs within these newly identified monophyletic groups [1].

Although EST distribution analysis in cDNA libraries from different poplar tissues may be indicative of their expression profile, the expression pattern of *PtLIM *genes has not been investigated. Using EST and microarray data, some poplar *LIM *genes (e.g. *PtWLIM1a *and *b *and *PtXLIM1a *and *b*) appear highly expressed in wood or in tension wood [1,14,15]. In trees, the formation of wood (or secondary xylem) is an important developmental process involving different steps: firstly cell division from the cambial meristem followed by xylem differentiation that include cell expansion, secondary wall synthesis, lignification and finally programmed cell death [16]. Produced in response to mechanical stresses, tension wood is a peculiar cellulose-rich and poorly lignified wood characterized by specific mechanical and structural properties that allow the tree to re-orientate its axis [17].

In this article, the expression of the twelve poplar *PtLIM *genes was investigated in different reproductive and vegetative tissues. Some *PtLIM *genes were ubiquitously expressed whereas others showed a preferential expression in pollen, cottony hairs or secondary xylem. Interestingly a subset of *PtLIM *genes was up-regulated in tension wood area and all duplicated genes exhibited distinct expression pattern suggesting subfunctionalisations. From this expression analysis and considering the previous phylogenetic classification we suggest a simplified nomenclature for the poplar LIM domain family.

## Findings

### Expression of poplar *LIM *genes in vegetative and reproductive organs

Previous EST distribution analyses indicated that *PtXLIM1a *and *b *and *PtWLIM1a *and *b *transcripts were mostly found in wood forming tissues whereas *PtWLIM2a *and *b *genes seemed to be ubiquitously expressed [1]. However, only a few *PtLIM1a *and *b *ESTs were found in undifferentiated cells and vegetative buds cDNA libraries. Moreover, *PtPLIM2a, b, c *and *d *transcripts were not or only poorly represented in poplar cDNA libraries preventing any inference on their expression profile. Therefore, the expression level of the twelve *PtLIM *genes was measured in different vegetative and reproductive tissues by semi quantitative RT-PCR.

**Figure 1:**
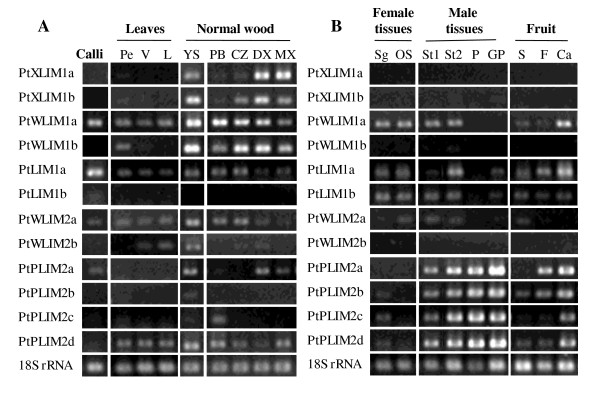
**Expression of *PtLIM *genes using semi quantitative RT-PCR analysis in poplar trees**. **A**. Vegetative organs: Three straight young poplars (*Populus tremula *× *P. alba*) producing normal wood were cultivated in the greenhouse for three months. Phloem and bark (PB), cambial zone (CZ), developing xylem (DX) and mature xylem (XM) were scraped from the stem. To study primary growth, young green portions of stems (YS) were collected just below the apex. Leaf vascular tissues like petioles (Pe) and main vessels (V) were separated from limbs (L). Two-weeks-old dark-grown calli (C) were also harvested. **B**. Reproductive organs: female flowers were dissected from mature poplar trees (*Populus nigra*) growing in the nursery and the stigma (Sg) were separated from the ovary and style (OS). Two different developmental stages were analyzed on male flowers: partially-open flowers (St1) at an early developmental stage, and fully-open flowers at pre-anthesis stage (St2). Mature pollen (P) and germinated pollen (GP) were also analyzed, as well as seeds (S) separated from both the cottony fibers (F) and the capsule protecting seeds (Ca). *18S rRNA *signals are indicative of the total RNA quantity in each sample

The *PtXLIM1a *and *b *and *PtWLIM1a *and *b *genes were strongly expressed in poplar stem particularly in wood, whatever the age of the tree (Figure 1 and Additional file 1). Moreover, they also showed a strong expression in the root differentiating xylem. These genes, except *PtWLIM1a*, were not or only weakly expressed in leaves, calli, and reproductive organs indicating a preferential expression in vascular tissues. *PtXLIM1a *expression appeared higher in developing and mature xylem (DX and MX) than in cambial zone (CZ) and phloem, suggesting specificity toward secondary xylem differentiation. Its duplicated gene *PtXLIM1b *as well as *PtWLIM1b *were more expressed in DX than in MX both in stem and root. Although the expression of *PtWLIM1a *gene was stronger in the stem, it seemed to be widely expressed in the whole tree except in pollen. From these results, genes from the XLIM1 and WLIM1 subgroups may play an important function during wood formation in poplar.

The duplicated *PtLIM1a *and *b *genes displayed distinct expression patterns. Whereas *PtLIM1a *gene was expressed in a wide range of tissues, the *PtLIM1b *gene appeared specifically expressed in reproductive organs including fruit, male and female flowers and to a lesser extent pollen (Figure 1). Interestingly, like *PtWLIM1a, PtLIM1a *expression is the highest in calli as well as in cottony hair (F). The expression pattern of the duplicated *PtWLIM2a *and *b *genes were strikingly different although their expression appeared rather weak in most poplar tissues. Indeed, *PtWLIM2a *was ubiquitously expressed in the plant when *PtWLIM2b *was mostly expressed in leaf and stem tissues. The *PtWLIM1a, PtLIM1a *and *PtWLIM2a *and *b *genes were also expressed in young roots collected from *in vitro *poplar plantlets, indicative of a preferential expression in tissues at primary growth stage (data not shown). Thus, *PtLIM1a, PtWLIM1a *and *PtWLIM2a *appeared widely expressed in poplar whereas the expression of their respective duplicated genes seemed more restricted to particular tissues.

*PtPLIM2a *and *b *exhibited a very strong expression in stamens, mature pollen and especially in germinated pollen. Interestingly, the duplicated *PtPLIM2c *and *d *genes, closely related to *PtPLIM2a *and *b*, were also highly expressed in the same tissues. The rather weak expression of these four genes in stamens at an early developmental stage compared to older stamens, pollen and germinating pollen suggests an expression in microspores rather than in filament, anther wall, rachis or peduncle supporting stamens. Surprisingly, these four genes were also highly expressed in isolated cottony hairs and in the capsules bearing these fibers, indicative of a dual expression in both pollen and cotton fibers. The *PtPLIM2a *gene was also expressed, but to a lower extent, in DX and MX, and *PtPLIM2d *transcript was detected in all parts of leaves and stems. These results indicate that *PtPLIM2a *and *b *genes and their paralogs *PtPLIM2c *and *d *may have redundant functions during pollen tube growth as well as in cotton fiber development.

### Comparison of poplar *LIM *gene expression between opposite and tension wood

Previously, *PtXLIM1a *ESTs were shown to be more abundant in tension wood DX than in opposite wood DX [1,15] and microarray analyses indicated a higher expression of *PtXLIM1b *in tension wood than in normal wood [14]. This prompted us to investigate more precisely the expression pattern of the *PtLIM *genes during tension wood differentiation on artificially tilted poplar trees. Tension wood, formed at the upper side of the tilted stem, is easily recognizable as a bright whitish crescent, and its presence was confirmed by the amplification of *PopFLA1 *gene (data not shown) a marker of tension wood formation [18].

**Figure 2:**
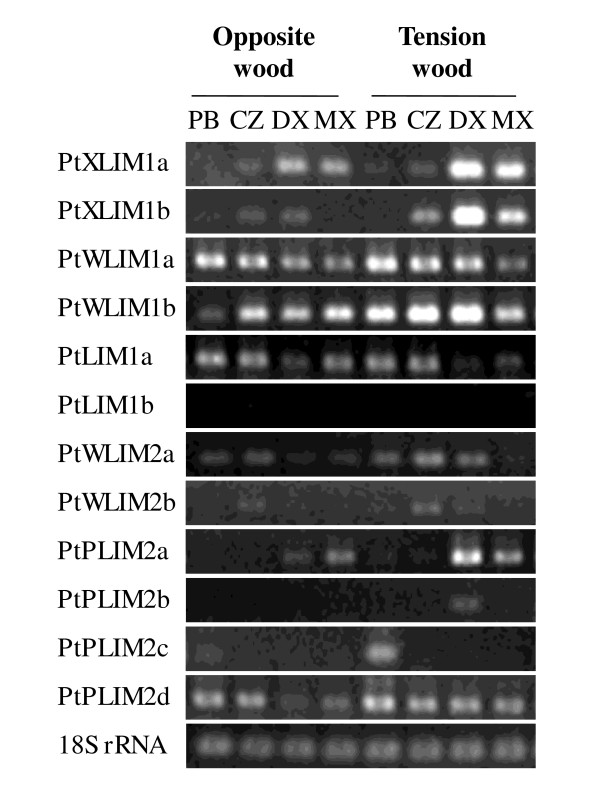
**Expression of *PtLIM *genes using semi quantitative RT-PCR analysis in opposite and tension wood**. Three young poplars (*Populus tremula *× *P. alba*) were cultivated in the greenhouse for two months, and tilted for one month to induce tension wood formation. Phloem and bark (PB), cambial zone (CZ), developing xylem (DX) and mature xylem (XM) samples were scraped from the upper side (tension wood) and the lower side (opposite wood) of tilted stems. *18S rRNA *signals are indicative of the total RNA quantity in each sample

Unexpectedly, the semi quantitative RT-PCR results showed that a number of poplar *PtLIM *genes: *PtXLIM1a *and *b*, but also *PtWLIM1b, PtWLIM2a, PtPLIM2a *and *PtPLIM2d *exhibited a higher expression in tension wood compared to opposite wood (Figure 2). These results confirmed the increased accumulation of *PtXLIM1a *and *b *transcripts in DX from tension wood but also revealed a high expression level in tension wood MX. *PtPLIM2a *expression in tension wood appeared to be restricted to DX and MX. By contrast, the accumulation of *PtWLIM1b *transcript in tension wood was mainly localized in DX, CZ and phloem/bark. The accumulation of *PtWLIM2a *and *PtPLIM2d *in DX was only slightly higher in tension wood compared to opposite wood and the other *PtLIM *genes did not exhibit any tension wood preferential up-regulation. The induction of *PtXLIM1a *gene in tension wood was supported at the protein level by western blot analysis using purified anti-6His-PtXLIM1a antibodies. A 29 kDa polypeptide corresponding to the expected size of the PtXLIM1a protein was detected from the CZ to the MX but not in leaves and reproductive organs (Additional file 2, data not shown). The PtXLIM1a protein was particularly more expressed in DX from tension wood compared to opposite wood. In summary, *PtXLIM1a *and *b *as well as *PtWLIM1b *and *PtPLIM2a *are unambiguously tension wood-induced genes and their expression appears to fluctuate depending on xylem differentiation.

## Discussion

### Expression of *LIM *duplicated genes in poplar, toward a simplified nomenclature

The previous classification and nomenclature of plant LIM domain protein was complex because of the lack of expression data concerning genes belonging to newly identified XLIM1, PLIM2-like subgroups and βLIM1 group [1]. Furthermore, the expression of poplar genes from WLIM1, WLIM2 and PLIM2 subgroups was predicted from EST analysis and phylogenetic comparison. Thus, the expression pattern of *PtLIM *genes was analyzed on an exhaustive set of poplar tissues covering most of the tree developmental stages. From our results, the 12 *PtLIM *genes were all expressed in poplar indicating that pseudogenization events did not occur. Poplar *PtLIM *genes are organized in six pairs of duplicated genes, and the high sequence similarity between duplicated *PtLIM *genes, ranging from 85 to 95% of amino acid identity, suggests that they share similar functions [1]. However, a few pair of genes exhibit distinct expression patterns suggesting a subfunctionalisation in specific tissues or possibly a neofunctionalisation [19]. For example, the *PtWLIM1a *and *PtLIM1a *genes almost constitutively expressed may be involved in a general cellular function, whereas the corresponding duplicated genes probably evolved towards specific functions such as xylem development for *PtWLIM1b *and eventually reproductive processes for *PtLIM1b*. The finding that *PtXLIM1a *and *b *genes were preferentially expressed in xylem strengthens their classification into the XLIM1 monophyletic subgroup distinct from the WLIM1 subgroup (Additional file 3) [1]. *XLIM1 *genes probably result from an older duplication of the ancestral *WLIM1 *gene with a further specialization towards new vascular specific functions potentially important for tree physiology. From our results, we have now strong evidence supporting a pollen preferential expression for the *PtPLIM2c *and *d *genes classified into the PLIM2-like subgroup phylogenetically close to the pollen specific PLIM2 subgroup (Additional file 3) [1]. It is thus likely that orthologous genes from sunflower and tobacco classified within the PLIM2-like subgroup also share the same pollen preferential expression. According to their expression profile and phylogeny, we propose a new nomenclature for the poplar LIM domain protein family (Table 1). For simplification and clarity, the formerly named *PtβLIM1a *and *b, PtδLIM2a *and *b *and *PtGLIM1a *and *b *genes were renamed *PtLIM1a *and *b, PtPLIM2c *and *d *and *PtXLIM1a *and *b *respectively. This revised and more consistent gene nomenclature taking into consideration the expression pattern of poplar *LIM *gene could serve as a basis for gene naming in other plant species and will hopefully improve communication among scientists working on the LIM domain family.

**Table 1:** Phylogenetic classification and nomenclature of poplar *PtLIM *genes

Group	Subgroup	Poplar	Arabidopsis	Rice	Sunflower	Expression profile
αLIM1	XLIM1 (FLIM1)	PtXLIM1a (PtGLIM1a) PtXLIM1b (PtGLIM1b)	not found	not found	not found	secondary xylem
	
	WLIM1	PtWLIM1a PtWLIM1b	AtWLIM1	OsWLIM1	HaWLIM1	ubiquitous
	
	PLIM1	not found	not found	not found	HaPLIM1a-SF3 HaPLIM1b	pollen

βLIM1	βLIM1	PtLIM1a (PtβLIM1a) PtLIM1b (PtβLIM1b)	not found	not found	HaLIM1 (HaβLIM1)	ubiquitous

γLIM2	WLIM2	PtWLIM2a PtWLIM2b	AtWLIM2a AtWLIM2b	OsWLIM2	HaWLIM2	ubiquitous

δLIM2	PLIM2	PtPLIM2a PtPLIM2b	AtPLIM2a AtPLIM2b AtPLIM2c	OsPLIM2a OsPLIM2b OsPLIM2c	HaPLIM2a	pollen
	
	PLIM2-like (δLIM2)	PtPLIM2c (PtδLIM2a) PtPLIM2d (PtδLIM2b)	not found	not found	HaPLIM2b (HaδLIM2a) HaPLIM2c (HaδLIM2b)	pollen

The old nomenclature of *LIM *gene and phylogenetic subgroups [1] is indicated under bracket

### Expression pattern of *LIM *genes in differentiating secondary xylem and tension wood

According to the expression study, the *PtXLIM1a *and *b *and *PtWLIM1b *genes were preferentially expressed in differentiating secondary xylem both in root and shoot, and were up-regulated in tension wood (Figures 1 and Figure 2, Additional file 1). Wood formation involves cell expansion and deposition of a highly lignified secondary cell wall [15,16]. The tobacco NtWLIM1 ortholog probably acts as a transcription factor activating the expression of *PAL, 4CL *and *CAD *genes coding for enzymes of the lignin biosynthesis pathway [5]. Interestingly, this protein also acts on the stabilization of actin bundles [6]. Actin cables, longitudinally oriented in vessels and fibers, certainly play an essential role in the cytoplasmic streaming and the distribution of cellulose and lignin precursors throughout these very elongated cells [20]. Taken together, both expression patterns and potential functions suggest the involvement of *PtXLIM1a *and *b *and *PtWLIM1b *in the differentiation of xylem cells, during cell elongation and secondary cell wall formation. Although some variations do exist, the common up-regulation of several *PtLIM *genes in tension wood may be indicative of a functional redundancy during tension wood formation. We know that tension wood is enriched in cellulose and contains less lignin than opposite or normal wood [17], and in accordance, the expression of *PAL, 4CL *and *CAD *genes involved in lignin biosynthesis is lower in poplar tension wood [14]. However, this merely applies during the deposition of the cellulose rich gelatinous layer (G-layer), specific to tension wood fibers, that is possibly devoid of any lignin, whereas the sub layers (S1 and S2) deposited before the G-layer contains regular amount of lignin [21]. Therefore, we cannot rule out that poplar LIM domain proteins act as transcriptional activators of lignin biosynthetic genes as proposed for the NtWLIM1 protein [5]. Interestingly, it was demonstrated that muscle specific CRP2 and CRP3 proteins are involved in the stretch response and the regulation of cell contractile force by interacting with actin stress fibers [22,23]. The actin binding and bundling functions between plant LIM domain proteins and the related animal CRP proteins is highly conserved [6-9]. According to the high expression of several *PtLIM *genes in tension wood, it is tempting to speculate that poplar LIM proteins may also be implicated in the perception/transduction of mechanical stresses through interactions with actin cables just as proposed for CRP in muscle cells [3,22]. However, such an involvement of actin bundles in tensile or contractile strain still needs to be demonstrated in tree wood fibers.

### Plant LIM domain proteins and anisotropic cell elongation

Poplar *PLIM2 *genes are preferentially expressed in mature and germinating pollen. These genes are potentially involved in pollen tube growth as already suggested for their orthologs in tobacco, sunflower and *Arabidopsis*. Indeed, *NtPLIM1, NtPLIM2, HaPLIM2, AtPLIM2a, b *and *c *genes are highly expressed in pollen and HaPLIM1-SF3 protein strongly accumulates in germinating pollen cones [2,8,13]. Poplar *PLIM2 *genes together with the *PtWLIM1a *and *PtLIM1a *genes were also strongly expressed in cottony fibers and capsules. Poplar cottony fibers resemble to cotton fibers from *Gossypium sp*. even though they originate from placenta and are borne by the capsule base [24]. Cotton fibers are unicellular trichomes composed of 95% cellulose that undergo considerable cell elongation with thick longitudinally oriented actin filaments [25,26]. Remarkably a high number of EST homologous to LIM domain protein has been detected in cotton fibers libraries [1]. Both our data and previous studies indicate that most of the plant *LIM *genes are expressed in cells with anisotropic elongation, such as xylem fibers, pollen tubes, and cottony fibers. This expression pattern is in agreement with the capacity of plant LIM proteins to bind and bundle actin filaments in order to favor the formation of thick actin cables [6,8,9]. Indeed longitudinally-oriented thick actin cables may be very important for effective tip growth and anisotropic cell expansion. Further genetics, biochemical and cell biological studies are required to characterize the function of plant LIM domain protein during cell elongation.

## Methods

### Sampling of poplar tissues

Vegetative tissues have been harvested on *Populus tremula *x *P. alba*, INRA clone 717-1-B4, poplar trees. Coppiced poplar trees kept at 4°C during the winter were transferred mid-March in the greenhouse, potted in compost (3 L) and individually supplied with water and fertilizers by a drip system. Straight stems and leaves were collected on three individual 3 month-old young poplar trees. Differentiating xylem (DX) and mature xylem (MX) samples were sequentially scraped from the debarked stem with a scalpel (Additional file 4). Cambial zone (CZ) was first lightly scraped from the peeled bark, while the remaining tissues consisted of the phloem and bark (PB) samples. Young stem (YS) samples enriched in primary growth tissues were the green stem portions collected below the apex. Following the sampling procedure described above, tension and opposite wood samples were collected, respectively at the upper and lower sides of the stems of three trees tilted for one month. Petiole (Pe) and main vessels (V) were separated from the limb (L) of leaves with a scalpel.

A number of samples were also collected during the spring, when cambium was very active, on a four year-old mature poplar tree cultivated in the tree nursery. CZ samples were lightly scraped from the peeled bark of the trunk. Then, phloem (Ph) samples were peeled from bark (B) samples. DX samples were first gently scraped from the debarked stem, while MX was sampled as wood chips from the remaining stem. The different samples from the main root were harvested following the same procedure, except that the peeled cortex contains both secondary phloem and root epidermis (PE), and after DX removal, the root MX consisted of the entire central cylinder including the pith. Undifferentiated cells were collected from calli (INRA clone 717-1-B4) cultivated for 2 weeks at 24°C in the dark on MB5 agar plates [27].

Because *Populus tremula *x *P. alba *trees from INRA 717-1-B4 clone were female hybrid clones, female and male flowers have been collected on *Populus nigra *and *P. nigra *var. *Italica *poplar trees. Excised branches bearing immature male or female flower catkins from trees growing on the Loire riverside were harvested in early April 2006. Female and male branches were respectively transferred in compost and water. One month later, after anthesis, the rachis, peduncles and bracts were removed from female flowers with a forceps and a scalpel. The stigmas (Sg) were separated from the ovaries and styles (OS) (See Additional file 4). After removal of protecting calyx, stamens with peduncles and rachis were collected from male flowers at early (St1) or late (St2) developmental stages. St1 corresponded to young and small partially-open male flowers, bearing sessile stamens on the rachis, whereas at St2, just before anthesis, male flowers were fully expanded and bore elongated rachis and peduncles with spreader stamens. Mature pollen (P) was spread on modified MS/2 media [28,29] and germinated pollen (GP) with elongated pollen tubes was harvested 24 h after germination. In order to produce and collect seeds, female flowers were manually fecundated with a paintbrush; one month later, the cottony hairs or fibers (F) were separated from seeds (S) and capsules (Ca).

Samples were immediately frozen upon collection, ground in liquid nitrogen and stored at -80°C before use. Samples collected from leaves and stem of 3 different greenhouse-grown poplar trees were pooled after grinding and before RNA extraction.

### RNA extraction and semi quantitative RT-PCR analyses

Total RNAs were prepared using the method described by Chang and colleague [30]. After overnight precipitation with LiCl, RNAs were further purified using QIAGEN RNEasy kit ^® ^according to manufacturer's instructions. RNA concentration and quality were determined by OD measurement at 260 nm and 280 nm. Two micrograms of total RNA were reverse transcribed using 500 ng of oligo(dT)12-18 and 200 units of SuperScript™ II RT in a total volume of 20 μl following the manufacturer's instructions (Invitrogen™, Life technologies). After first-strand cDNA synthesis, RNA complementary to the cDNA was removed by incubating the reaction with 2 units of *E. coli *RNase H at 37°C for 20 min. Gene-specific PCR primers were designed to amplify *PtLIM *genes in *Populus trichocarpa *Nisqually genotype as well as in *P. tremula × P. alba *(INRA clone 717-1-B4) when cDNA sequences (*PtXLIM1a *and *b, PtWLIM1a *and *b, PtWLIM2a *and *PtPLIM2a*) were available [1]. Primers were located at both ends of gene coding sequences in order to amplify the four introns and therefore detect any genomic contamination. PCR amplification was done with 1/60 of the first-strand cDNA as template, 0,5 unit of rTaq DNA polymerase (Invitrogen™, Life technologies), 1,25 mM MgCl_2 _and 0,2 μM of gene specific primers (Additional file 5) in a total volume of 20 μl. For each primer pair, gene-specific amplification was optimized and verified on available *PtLIM *cDNA clones used as template. For *PtLIM *genes, the cycling conditions were 94°C for 5 min for one initial step followed by 94°C for 45 s, 65 or 60°C for 45 s and 72°C for 1 min, for 25 to 40 cycles (See Additional file 5). For *18S rRNA*, the cycling conditions were 94°C for 5 min for one initial step followed by 94°C for 45 s, 55°C for 1 min and 72°C for 1 min 30, for 20 cycles [18]. The PCR was terminated with one extra step at 72°C for 10 min and hold at 4°C. The amplification was done using a GeneAmp^® ^PCR System 9700 (Applied Biosystems). Ten microliters of each PCR product were electrophoresed on a 1% agarose gel that was scanned under UV light after ethidium bromide staining. Results presented are the representative illustration of at least three independent semi quantitative RT-PCR experiments. The identity of each PCR product was checked by sequencing.

### Production of 6His-PtXLIM1a protein, protein extraction and Western blot

See Additional file 6 for detailed description of the procedure.

### Availability of supporting data

The data sets supporting the results of this article are included within the article and its additional files.

## Competing interests

The authors declare that they have no competing interests.

## Authors' contributions

DA participated in the conception and design of the study, carried out semi-quantitative RT-PCR experiments as well as the production and purification of recombinant protein, and wrote the manuscript. AD and J-CL participated in the design of the study, manuscript revising and provided helpful discussions. M-CL-D performed sequencing of PCR products. MV participated in the collection of reproductive samples. NB carried out protein extraction, antibodies purification and western blot. HB coordinated and contributed to production and purification of recombinant protein. GP coordinated the study, participated in its conception and design as well as revisions to the manuscript. All authors have read and approved the final manuscript.

## Supplementary Material

Additional file 1 **PtLIM genes expression in wood tissues of an adult poplar tree**. Expression of *PtLIM *genes using semi quantitative RT-PCR analysis in the trunk and main root of a four-year old poplar tree. Click here for file

Additional file 2 **Expression of PtXLIM1a protein in opposite and tension wood**. Immunodetection of PtXLIM1a protein in total protein extracts collected from tilted trunk of four-year old poplar trees. Click here for file

Additional file 3 **Phylogenetic tree of plant LIM domain proteins**. Phylogenetic tree of poplar, Arabidopsis, rice, tobacco and sunflower LIM domain proteins. Click here for file

Additional file 4 **Sampling of wood tissues and reproductive organs**. Illustration of samples collected from wood forming tissues on stems and description of male and female flowers used in the study. Click here for file

Additional file 5 **Primers used in semi-quantitative RT-PCR**. Forward and reverse primer sequences (5' to 3') for all *PtLIM *genes analyzed and PCR conditions used for semi-quantitative RT-PCR. Click here for file

Additional file 6 **Supplemental methods for protein extraction and western blot**. Method used for protein extraction, western-blot analysis, production and purification of 6His-PtXLIM1a recombinant protein. Click here for file
