# Supplementary material for: Expression analysis of LIM gene family in poplar, toward an updated phylogenetic classification
Source: BMC Res Notes. 2012 Feb 17;5:102. doi: 10.1186/1756-0500-5-102 (PMC3392731; doi:10.1186/1756-0500-5-102)
Supplement: Additional file 2 — Expression of PtXLIM1a protein in opposite and tension wood. Immunodetection of PtXLIM1a protein in total protein extracts collected from tilted trunk of four-year old poplar trees. [file 1756-0500-5-102-S2.PDF]

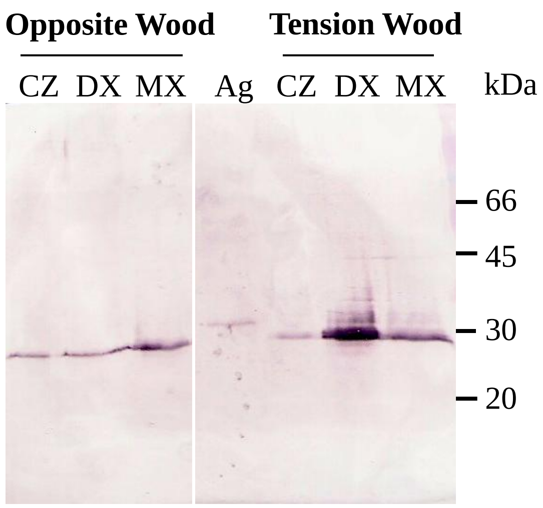

Additional file 2 - Expression of PtXLIM1a protein in opposite and tension wood

The expression of PtXLIM1a protein was analyzed by western blot on total proteins extracted from different tissues sampled on the stems of 3-month old poplar trees (*Populus tremula* *P*. *alba*) tilted for 1 month. Cambial zone (CZ), developing xylem (DX) and mature xylem (XM) were scraped from the upper side (tension wood) and the lower side (opposite wood) of tilted stems. Thirty µg of total protein or 500 ng of purified 6His-PtXLIM1a recombinant protein (Ag) were loaded onto a SDS-PAGE gel. Equal loading of proteins was verified after transfer onto membrane by red Ponceau staining (data not shown). In gel purified anti-6His-XLIM1a antibodies were diluted to 1/500 and the membranes were revealed by colorimetric detection using the NBT/BCIP substrates. The weight of molecular markers is indicated in kDa.
