# Supplementary material for: Expression analysis of LIM gene family in poplar, toward an updated phylogenetic classification
Source: BMC Res Notes. 2012 Feb 17;5:102. doi: 10.1186/1756-0500-5-102 (PMC3392731; doi:10.1186/1756-0500-5-102)
Supplement: Additional file 3 — Phylogenetic tree of plant LIM domain proteins. Phylogenetic tree of poplar, Arabidopsis, rice, tobacco and sunflower LIM domain proteins. [file 1756-0500-5-102-S3.PDF]

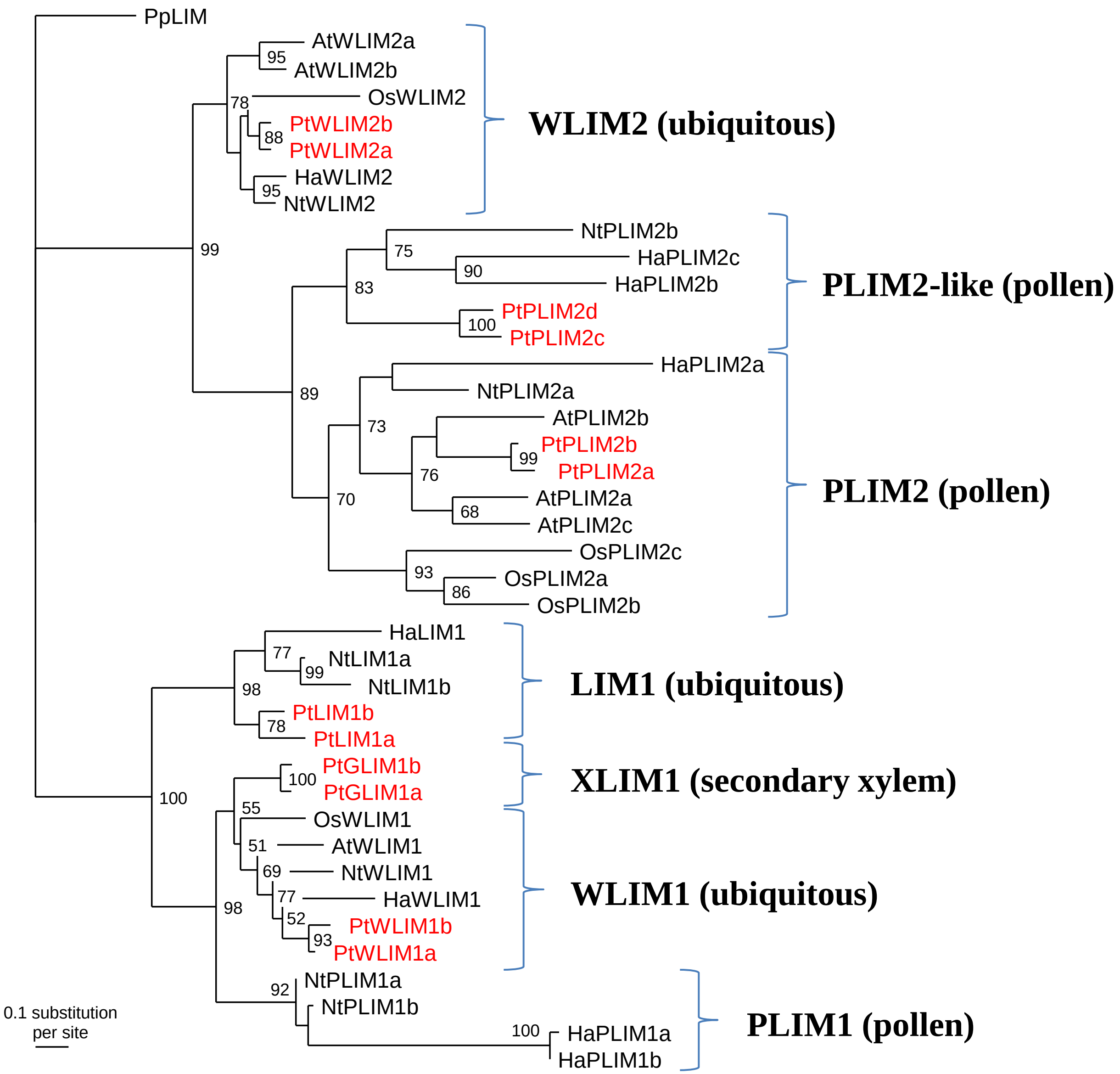

Additional file 3 - Phylogenetic tree of plant LIM domain proteins

Amino acids sequences of poplar, Arabidopsis, rice, tobacco and sunflower LIM domain proteins were aligned with ClustalW and have been analyzed by the maximum likelihood method using the Phyml and BIONJ programs (Arnaud et al., 2007). The numbers at the nodes represent bootstraps values (≥50) based on 100 replications. Poplar PtLIM genes are highlighted in red. A species acronym is added before each LIM protein name: Pt, *Populus trichocarpa*; At, *Arabidopsis thaliana*; Os, *Oryza sativa*; Ha, *Helianthus annuus*; Nt, *Nicotiana tabacum*; Pp, *Physcomitrella patens*.
