# Supplementary material for: Expression analysis of LIM gene family in poplar, toward an updated phylogenetic classification
Source: BMC Res Notes. 2012 Feb 17;5:102. doi: 10.1186/1756-0500-5-102 (PMC3392731; doi:10.1186/1756-0500-5-102)
Supplement: Additional file 4 — Sampling of wood tissues and reproductive organs. Illustration of samples collected from wood forming tissues on stems and description of male and female flowers used in the study. [file 1756-0500-5-102-S4.PDF]

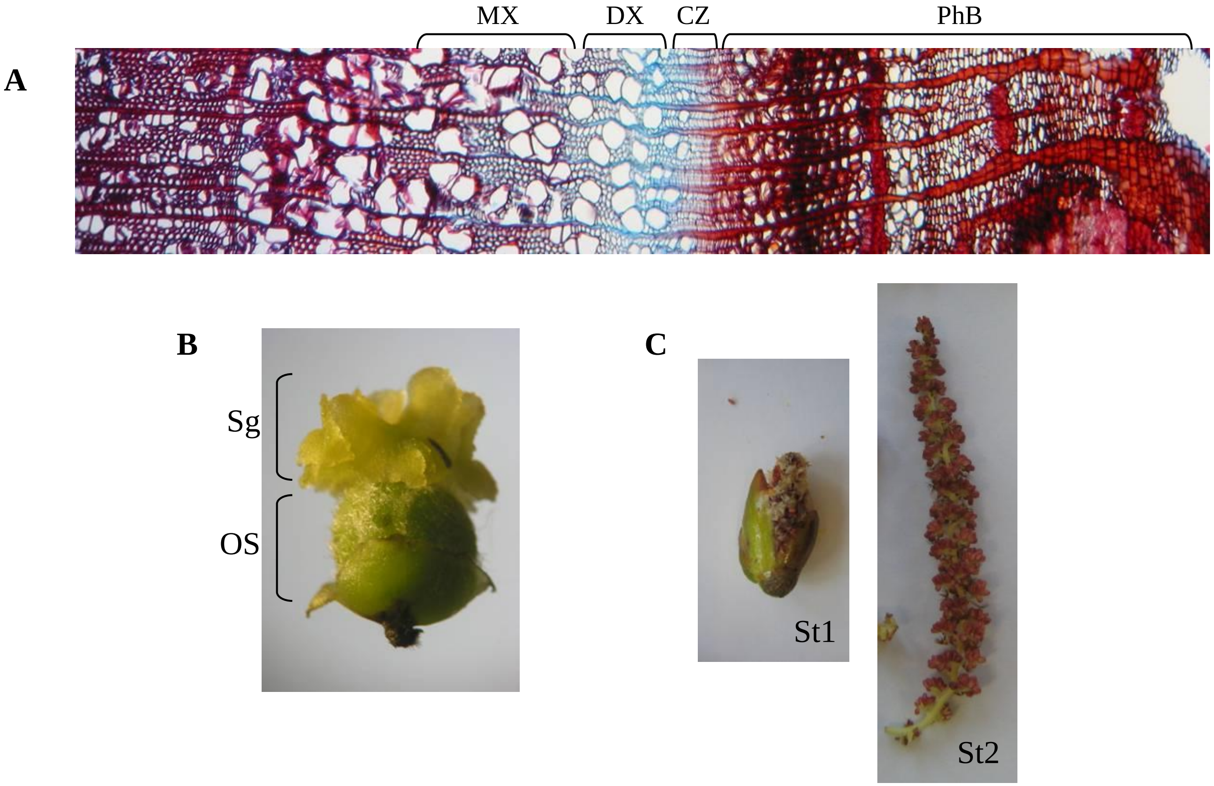

Additional file 4 - Sampling of wood tissues and reproductive organs.

A. Safranin-Astra blue staining of a cross section from opposite wood at the lower side of tilted stem (photo Laurans F., INRA Orléans). Young poplar trees (*P*. *tremula*   *P*. *alba*) were cultivated in a greenhouse. Lignified cell walls appear in red. The different samples collected are the phloem and bark (PhB), the cambial zone (CZ), the developping xylem (DX), and the mature xylem (MX), see Material and Methods.

B. On female flowers after anthesis, stigma (Sg) was separated from ovary and style (OS) that also included the perianth located at the basis of the carpel.

C. Male flowers at two developmental stages: St1 represents an early stage where male catkins are small, partially opened with sessile stamens. St2 corresponds to a late developmental stage just before anthesis with elongated and hanging catkins bearing spread stamens on the rachis.
