# Supplementary material for: Expression analysis of LIM gene family in poplar, toward an updated phylogenetic classification
Source: BMC Res Notes. 2012 Feb 17;5:102. doi: 10.1186/1756-0500-5-102 (PMC3392731; doi:10.1186/1756-0500-5-102)
Supplement: Additional file 5 — Primers used in semi-quantitative RT-PCR. Forward and reverse primer sequences (5' to 3') for all PtLIM genes analyzed and PCR conditions used for semi-quantitative RT-PCR. [file 1756-0500-5-102-S5.PDF]

Additional file 5 – Primers used in semi-quantitative RT-PCR

The specificity of each couple of primers was verified by sequencing the PCR products for all *PtLIM* genes.

Gene	Size of PCR products (bp)	Tm (°C)	Number of cycles	Forward and Reverse primer sequences
PtWLIM1a	634	65	25	GACAAGTGTTTCGGCTGCAAGAAC<br>AGGCAGGAAAATGCAGGATGAACA
PtWLIM1b	625	65	30	GACAAGTGTTTCGGCTGCAAGAAC<br>GCAAGGCAGGAAAATACACTCGTT
PtXLIM1a	590	65	30	CCTGTTGATAATGAGAACGCCAGC<br>CAGCAATCTCTACAGCAGTGATGC
PtXLIM1b	583	65	30	CCTGTTGATAATGAGAACGCAAGC<br>CACGGCGGTGATATTCACAGTG
PtLIM1a	444	60	35	TTAGATGCCACCACTGCAAG<br>CTGGTGTAACATGTTCGTGCT
PtLIM1b	436	60	35	TTAGATGCCACCACTGCAAG<br>AGATGTTCATGTTTGCCGAA
PtWLIM2a	646	65	30	GGTGATCCGAGACAGGTAAACAGT<br>GAGATGTTTAAGCCTCTGGCACAG
PtWLIM2b	639	65	40	TCCAAAGGAGTTAACAGGACAGTC<br>GAGATGTTTAAGCCTCTGGCACAG
PtPLIM2a	609	65	35	CATTCACGGGAACCCTGGATAAG<br>TGCTCTTCTGACTTTTCCTCCTCC
PtPLIM2b	562	65	40	CATTCACGGGAACCCTGGATAAG<br>TCGGCCAGTTCAGGAGGTGG
PtPLIM2c	434	60	35	GGACCTTAGAGAAATGCAAGG<br>GATACCATCAAGAGCTGCATAA
PtPLIM2d	426	60	35	GGACCTTAGAGAAATGCAAGG<br>CAAGAGCAGCGTAGGATGAT
18SRNA	232	55	20	CTTCGGGATCGGAGTAATGA<br>GCGGAGTCCTAGAAGCAACA
