# Supplementary material for: Expression analysis of LIM gene family in poplar, toward an updated phylogenetic classification
Source: BMC Res Notes. 2012 Feb 17;5:102. doi: 10.1186/1756-0500-5-102 (PMC3392731; doi:10.1186/1756-0500-5-102)
Supplement: Additional file 6 — Supplemental methods for protein extraction and western blot. Method used for protein extraction, western-blot analysis, production and purification of 6His-PtXLIM1a recombinant protein. [file 1756-0500-5-102-S6.PDF]

Additional file 6 - Supplemental methods for protein extraction and Western blot

To produce the 6His-PtXLIM1a recombinant protein in *E. coli*, the *PtaXLIM1a* coding sequence was cloned into the pENTR™/D-TOPO® (Invitrogen™) and then recombined into pDEST17 (Invitrogen™) using the Gateway system (Fisher Scientific Bioblock, Illkirch, France). The resulting plasmid construct was transferred into the BL21 pLysS bacterial strain and the production of 6His-PtXLIM1a recombinant protein was induced by 0,1 mM IPTG. 6His-PtXLIM1a recombinant protein was purified as inclusion bodies followed by immobilized cobalt affinity chromatography "BD TALON™ Metal Affinity Resin". Antibodies against the 6His-PtXLIM1a protein were produced by AGRO-BIO (La Ferté St Aubin, France). Two rabbits were immunized by five intra-dermal injections at 0, 14, 28, 42 and 56 days and the immune serum was obtained after 77 days. Anti-6His-PtXLIM1a antibodies were purified by blot affinity purification on SDS-PAGE gel against the purified 6His-PtXLIM1a recombinant protein according to [1].

Total proteins were extracted using the TCA-acetone method [2]. Briefly, proteins from 200 to 300 mg of ground plant material were precipitated for 1 h at –20°C by an acetone-TCA mixture (9:1) containing 0.07% β-mercaptoethanol. After 30 min centrifugation at 16000 *g*, the protein pellet was washed three times with acetone containing 0.07% β-mercaptoethanol and dried under vacuum. Before storage at -20°C, the proteins were dissolved in the loading buffer, containing 80 mM Tris-HCl pH 6.8, 0.1 M DTT, 4 M urea, 2% SDS, and 10% glycerol, followed by a 15 min centrifugation at 16000 *g* to remove the insoluble cell debris. The quantity of soluble proteins was estimated by the Bradford method [3]. After boiling for 5 min, 30 µg of soluble proteins were separated by SDS-PAGE on 12% polyacrylamide gels using an acrylamide/bisacrylamide ratio of 37.5:2.5 and Tris-glycine-SDS (25 mM Tris, 192 mM glycine, 0.1% SDS) as running buffer. Proteins were subsequently transferred onto nitrocellulose membrane using the iBlot® Dry Blotting System (Invitrogen). Western blot analyses were performed using blot-affinity purified anti-6His-XLIM1a antiserum and secondary antibodies bound to alkaline phosphatase (P.A.R.I.S). Both antibodies were diluted to 1:500 and labelling was revealed by colorimetric detection using the NBT/BCIP substrates (Promega).

Reference:

1. Tang W-JY: **Blot-Affinity Purification of Antibodies.** In *Methods in Cell Biology. Volume* 37: Academic Press; 1993: 95-104
2. Baltz R, Schmit A-C, Kohnen M, Hentges F, Steinmetz A: **Differential localization of the LIM domain protein PLIM-1 in microspores and mature pollen grains from sunflower.** *Sexual Plant Reproduction* 1999, **12:**60-65.
3. Bradford MM: **A rapid and sensitive method for the quantitation of microgram quantities of protein utilizing the principle of protein-dye binding.** *Analytical Biochemistry* 1976, **72:**248-254.
