# Supplementary material for: Expression analysis of LIM gene family in poplar, toward an updated phylogenetic classification
Source: BMC Res Notes. 2012 Feb 17;5:102. doi: 10.1186/1756-0500-5-102 (PMC3392731; doi:10.1186/1756-0500-5-102)

Additional file 1 - *PtLIM* genes expression in wood tissues of an adult poplar tree.

The expression of *PtLIM* genes in the trunk and main root of a straight four-year old field-grown poplar tree was evaluated by semi quantitative RT-PCR analysis. On the trunk, the bark (B) was separated from the phloem (Ph). The cambial zone (CZ), the developing and mature xylem (DX and MX) were scraped off with a scalpel. The developing secondary xylem (DX) was harvested from the main root with a scalpel while the remaining peeled root (after DX removal) constituted the mature xylem (MX) sample. The cambial zone (CZ) was collected on the inner face of the secondary phloem and epidermis (PE). *18S rRNA* signals are indicative of the total RNA quantity in each sample.

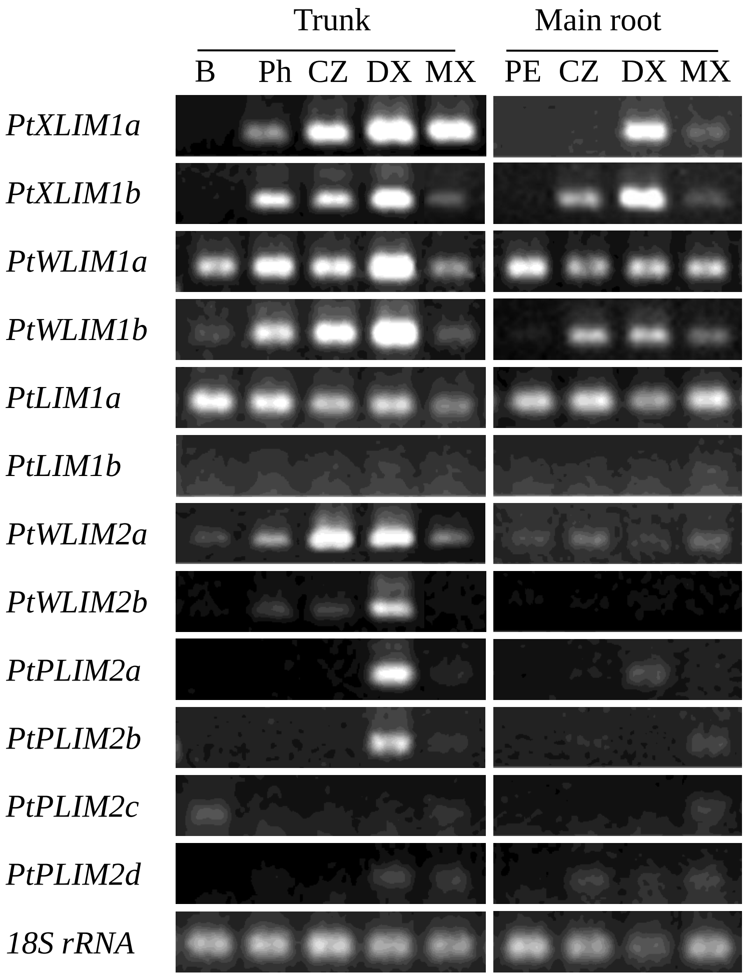

Supplement: Additional file 1 — PtLIM genes expression in wood tissues of an adult poplar tree. Expression of PtLIM genes using semi quantitative RT-PCR analysis in the trunk and main root of a four-year old poplar tree. [file 1756-0500-5-102-S1.PDF]
